# Preoperative pregabalin prolongs duration of spinal anesthesia and reduces early postoperative pain: A double-blind, randomized clinical CONSORT study: Erratum

**DOI:** 10.1097/01.md.0000503454.98968.e2

**Published:** 2016-10-07

In the article “Preoperative pregabalin prolongs duration of spinal anesthesia and reduces early postoperative pain: A double-blind, randomized clinical CONSORT study”,^[1]^ which appeared in Volume 95, Issue 36 of *Medicine*, Dr. Hyerim Lee's name was originally omitted from the list of authors. The article has since been corrected online.

## References

[1] Park M Jeon Y Preoperative pregabalin prolongs duration of spinal anesthesia and reduces early postoperative pain: A double-blind, randomized clinical CONSORT study. *Medicine.* 2016 95:e4828. 27603398 10.1097/MD.0000000000004828 PMC5023921

